# Comparison of diffusion-weighted imaging and contrast-enhanced T1-weighted imaging on a single baseline MRI for demonstrating dissemination in time in multiple sclerosis

**DOI:** 10.1186/1471-2377-14-100

**Published:** 2014-05-07

**Authors:** Chung-Ping Lo, Hung-Wen Kao, Shao-Yuan Chen, Chi-Ming Chu, Chia-Chun Hsu, Ying-Chu Chen, Wei-Chen Lin, Dai-Wei Liu, Wen-Lin Hsu

**Affiliations:** 1 Department of Radiology, Taichung Tzuchi Hospital, The Buddhist Tzuchi Medical Foundation, No 66, Sec 1, Fongsing Road, Taichung Tanzih District, 427, Taiwan; 2 School of Medicine, Tzu Chi University, Hualien, Taiwan; 3 Department of Radiology, Tri-Service General Hospital and National Defense Medical Center, Taipei, Taiwan; 4 Department of Neurology and Hyperbaric Medicine, Cardinal Tien Hospital, New Taipei City, Taiwan; 5 School of Medicine, Fu Jen Catholic University, New Taipei City, Taiwan; 6 Section of Biomedical Informatics, School of Public Health, National Defense Medical Center, Taipei, Taiwan; 7 Department of Neurology, Taichung Tzuchi Hospital, The Buddhist Tzuchi Medical Foundation, Taichung, Taiwan; 8 Department of Medical Imaging, Kaohsiung Medical University Hospital, Kaohsiung Medical University, Kaohsiung, Taiwan

**Keywords:** Brain, Demyelinating diseases, Diffusion magnetic resonance imaging, Gadolinium, Magnetic resonance imaging, Multiple sclerosis

## Abstract

**Background:**

The 2010 Revisions to the McDonald Criteria have established that dissemination in time (DIT) of multiple sclerosis (MS) can be demonstrated by simultaneous presence of asymptomatic gadolinium-enhancing and nonenhancing lesions on a single magnetic resonance imaging (MRI). However, gadolinium-based contrast agents (GBCAs) have contraindications. Diffusion-weighted imaging (DWI) can detect diffusion alterations in active inflammatory lesions. The purpose of this study was to investigate if DWI can be an alternative to contrast-enhanced T1-weighted imaging (CE T1WI) for demonstrating DIT in MS.

**Methods:**

We selected patients with clinically definite MS and evaluated their baseline brain MRI. Asymptomatic lesions were identified as either hyperintense or nonhyperintense on DWI and enhancing or nonenhancing on CE T1WI. Fisher’s exact test was performed to determine whether the hyperintensity on DWI was related to the enhancement on CE T1WI (*P* < 0.05). The sensitivity, specificity, positive predictive value (PPV), negative predictive value (NPV), and accuracy of the DWI to predict lesion enhancement were calculated.

**Results:**

Twenty-two patients with 384 demyelinating lesions that were hyperintense on T2-weighted imaging and more than 3 mm in size were recruited. The diffusion hyperintensity and lesion enhancement were significantly correlated (*P* <0.001). The sensitivity, specificity, PPV, NPV and accuracy were 100%, 67.9%, 32.3%, 100% and 72.1%, respectively.

**Conclusions:**

A hyperintense DWI finding does not necessarily overlap with contrast enhancement. There are many false positives, possibly representing other stages of lesion development. Although DWI may not replace CE T1WI imaging to demonstrate DIT due to the low PPV, it may serve as a screening MRI sequence where the use of GBCAs is a concern.

## Background

The diagnosis of multiple sclerosis (MS) is established on demonstration of central nervous system (CNS) demyelinating lesions with dissemination in space (DIS) and time (DIT) and exclusion of alternative diagnoses. The diagnosis can be made on clinical grounds alone, and magnetic resonance imaging (MRI) findings can replace objective clinical evidence of one lesion and one clinical attack once they meet MRI DIS and DIT criteria. The 2010 Revisions to the McDonald Criteria simplifies the MRI criteria, and the diagnosis of MS can be made based on a single MRI scan in some patients at the clinically isolated syndrome (CIS) stage [1]. According to criteria developed by Swanton et al., DIS can be demonstrated by more than one T2 lesion in at least two of four CNS areas affected by MS (periventricular, juxtacortical, infratentorial, and spinal cord) and DIT can be demonstrated by a new T2 and/or gadolinium-enhancing lesion on follow-up MRI or by simultaneous presence of asymptomatic gadolinium-enhancing and nonenhancing lesions at any time, representing demyelinating lesions in different stages of evolution (the latter proposed by Rovira et al.) [1-4].

Contrast enhancement in demyelinating lesions and blood–brain barrier (BBB) breakdown on histopathology have been generally regarded as signs of active perivascular inflammation. However, gadolinum-based contrast agents (GBCAs) have contraindications, such as increased risk for developing nephrogenic systemic fibrosis in patients with acute or chronic severe renal insufficiency (glomerular filtration rate below 30 mL/min/1.73 m^2^) and allergy to GBCAs [5,6]. Besides, in the 2013 Manual of Contrast Media, the American College of Radiology states that because it is unclear how GBCAs will affect the fetus, these agents should be administered with caution in pregnant women. They should only be used if their usage is considered critical and the potential benefits justify the potential risk to the unborn fetus [6]. In patients with contraindications or relative contraindications to GBCAs, an alternative MRI sequence may be needed for early and accurate diagnosis of MS. Diffusion-weighted imaging (DWI) has been widely used to diagnose acute ischemic infarction and also to detect diffusion alterations in active inflammatory lesions. Whether it can substitute for contrast-enhanced T1-weighted imaging (CE T1WI) to differentiate different features of demyelinating disease (e.g., DIT) has yet to be verified. The purpose of this study was to investigate the relationship of the signal intensity of demyelinating lesions on DWI to the status of enhancement on CE T1WI in baseline brain MRI in patients with clinically definite MS (CDMS).

## Methods

### Study population

From January 2001 to December 2012, patients who presented with acute CNS symptoms suggestive of demyelinating disease in our institutions were recruited in the cohort of CIS. Patients with final diagnosis of CDMS made by experienced neurologists after two or more clinical attacks of CNS demyelinating events and objective clinical evidence of two or more lesions as defined by the McDonald Criteria and exclusion of other possible alternative diseases (such as acute disseminated encephalomyelitis or neuromyelitis optica) were considered candidates for this study. The inclusion criteria also included: (1) age at onset between 15 and 50 years old; (2) patients’ baseline brain MRI studies performed within three months of symptom onset; (3) CE T1WI and DWI included in the MRI protocol; (4) no use of disease modifying drugs (e.g. interferon) or steroid before baseline brain MRI examinations to eliminate their effect on contrast enhancement and edema in demyelinating lesions. Ethical approval for patient recruitment in our previous study was extended to our present study by the institutional review board of Tri-Service General Hospital (TSGH IRB 1-101-05-004). Because this study was based on retrospective analysis of existing data, no additional approval was required.

### MRI sequences and imaging analysis

The MRI studies were performed on 1.5-T MR scanners. The brain MRI sequences included spin echo (SE) or fast spin echo (FSE) T1WI, T2-weighted imaging (T2WI), and T2-fluid-attenuated inversion recovery (T2-FLAIR) in the axial plane, T2WI or T2-FLAIR in the sagittal plane, and CE T1WI in the axial, coronal, and sagittal planes after injection of 0.1 mmol/kg of GBCAs. DWI was acquired with a single-shot echo planar spin-echo sequence in three orthogonal directions with a *b* value of 1000 sec/mm^2^ and a baseline image with a *b* value of 0 sec/mm^2^. Apparent diffusion coefficient (ADC) and exponential ADC (eADC) maps were automatically generated. The DWI was performed prior to administration of GBCAs with an identical slice thickness (5 mm) and position to T1WI, T2WI, and T2-FLAIR. The demyelinating lesions were first labeled by an experienced neuroradiologist on T2WI and T2-FLAIR with their sizes being no less than 3 mm for better delineation on CE T1WI and DWI. An experienced neurologist reviewed the medical records and then excluded the symptomatic lesions. The remaining demyelinating lesions were determined as either enhancing or nonenhancing on CE T1WI. On DWI, ADC and eADC maps, the signal intensity of the lesions was determined as either hyperintense, isointense or hyporintense to the surrounding normal-appearing white matter. The area of perilesional edema, if any, was not evaluated.

The demyelinating lesions were classified into four types according to their signal intensity on CE T1WI and DWI. Type I lesions were enhancing on CE T1WI and hyperintense on DWI. Type II lesions were enhancing on CE T1WI and nonhyperintense (isointense or hypointense) on DWI. Type III lesions were nonenhancing on CE T1WI and hyperintense on DWI. Type IV lesions were nonenhancing on CE T1WI and nonhyperintense on DWI. Each type was further divided into two subtypes according to the DWI, ADC and eADC. Subtype A lesions showed restricted water diffusion as hyperintense on DWI, hypointense on ADC and hyperintense on eADC. Subtype B lesions showed non-restricted water diffusion as iso- or hyperintense on ADC and iso- or hypointense on eADC. The DWI signal in subtype B lesions may be affected by the molecular motion of water and T2 shine-through effect and thus may be variable. The classification of the lesion types is summarized in Table 1. The foci on CE T1WI (either enhancing or nonenhancing) were taken as the gold standard for the diagnosis, and the signal intensity (hyperintense or nonhyperintense) of each lesion on DWI was compared to the status of enhancement on CE T1WI.

**Table 1:** Classification of the types and subtypes of demyelinating lesions

**Lesion type**	**CE T1WI**	**DWI**	**Lesion subtype**	**DWI**	**ADC**	**eADC**
I	C+	Hyper	A	Hyper	Hypo	Hyper
II	C+	Iso or hypo				
III	C–	Hyper	B	Iso, hypo or hyper (T2 T-S)	Iso or hyper	Iso or hypo
IV	C–	Iso or hypo				

CE T1WI, contrast-enhanced T1-weighted imaging; DWI, diffusion-weighted imaging; ADC, apparent diffusion coefficient; eADC, exponential apparent diffusion coefficient; C +, enhancing; C –, nonenhancing; hyper, hyperintense; iso, isointense; hypo, hypointense; T2 T-S, T2-shine through effect.

### Statistics

Fisher’s exact test was carried out to determine whether the hyperintensity of the lesions on DWI was related to contrast enhancement on CE T1WI. Significance was set at a *P* value of less than 0.05. The sensitivity, specificity, positive predictive value (PPV), negative predictive value (NPV), and accuracy of the DWI for prediction of the enhancement of demyelinating lesions on CE T1WI were also calculated. The patient numbers fulfilling DIT using CE T1WI or DWI were also calculated.

## Results

A total of 22 patients (M/F = 5/17) with CDMS ranging in age from 15 to 50 years (mean: 32.8 years, standard deviation: 10.2) fulfilled the inclusion criteria. The 22 patients had converted to CDMS with intervals ranging from 2 to 24 months after the onset of initial clinical events (mean: 8.5 months, standard deviation: 5.2). The patients’ average baseline expanded disability status scale (EDSS) was 2.7 (range: 1.0-5.0, standard deviation: 1.2). The interval between symptoms onset and baseline brain MRI examinations ranged from 1 to 44 days (mean: 18 days, standard deviation: 10.7). The 22 brain MRI examinations disclosed a total of 384 demyelinating lesions (51 enhancing and 333 nonenhancing) more than 3 mm in size on T2WI and T2-FLAIR (range: 1–78, mean: 17.5, standard deviation: 17.4).

**Figure 1:**
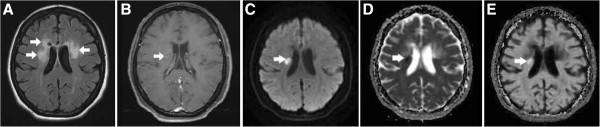
**A type IA lesion in a patient with MS. (A)** Axial T2-FLAIR brain MRI shows several demyelinating lesions in the bilateral periventricular white matter (arrows). **(B)** CE T1WI shows a small central nodular enhancement in one of the lesions on the right side (arrow). **(C–E)** The lesion (arrow) is hyperintense on the DWI, hypointense on the ADC map, and hyperintense on the eADC map, indicating an early stage of inflammation with restricted water diffusion (cytotoxic edema).

**Figure 2:**
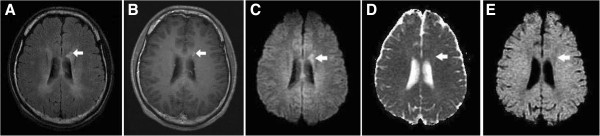
**A type IB lesion in a patient with MS. (A)** Axial T2-FLAIR brain MRI shows dirty appearance of the bilateral periventricular white matter and a demyelinating lesion on the left side (arrow). **(B)** The lesion (arrow) shows enhancement on the CE T1WI. **(C–E)** It is hyperintense on the DWI, isointense to slightly hyperintense on the ADC map and isointense to slightly hypointense on the eADC map. The appearances suggest an active stage of demyelination with conversion to vasogenic edema and the T2 shine-through effect.

**Figure 3:**
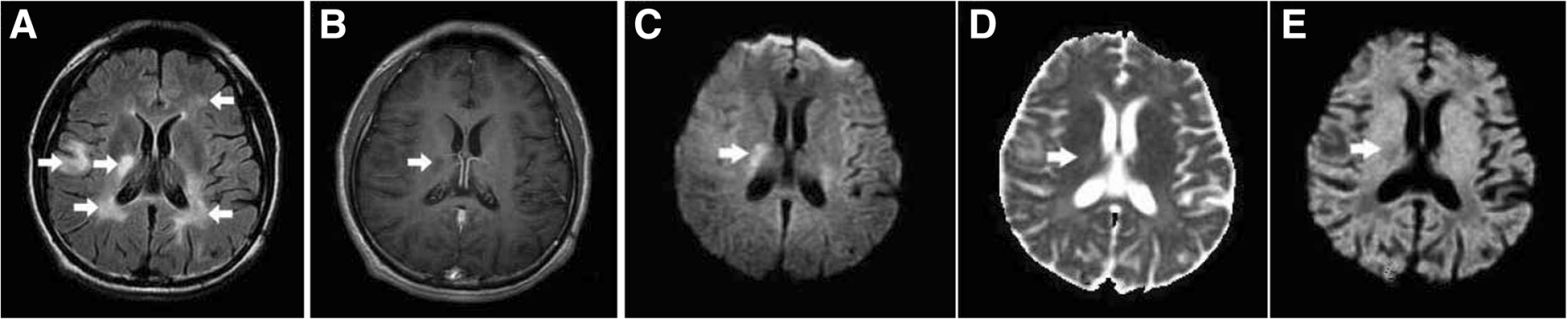
**A type IIIB lesion in a patient with MS. (A)** Axial T2-FLAIR brain MRI shows multiple demyelinating lesions (arrows) in the bilateral periventricular white matter and one in the right subcortical white matter. **(B)** All of the lesions show no enhancement on the CE T1WI, including one lesion involving the right periventricular white matter (arrow). **(C–E)** The lesion (arrow) is hyperintense on the DWI, hyperintense on the ADC map, and hypointense on the eADC map, indicating residual vasogenic edema and the T2 shine-through effect. The hyperintensity on the DWI disappeared on follow-up MRI three months later (not shown).

**Figure 4:**
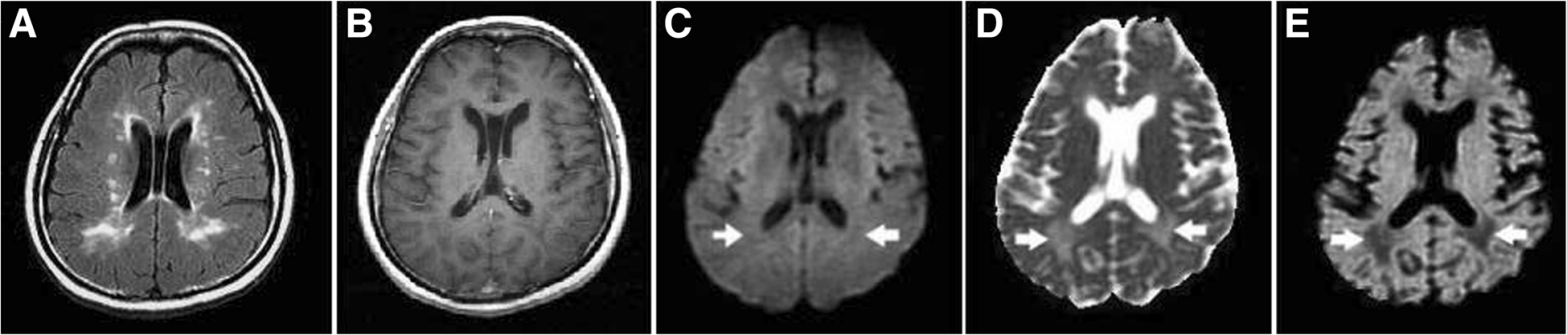
**Type IVB lesions in a patient with MS. (A)** Axial T2-FLAIR brain MRI shows multiple demyelinating lesions in the bilateral periventricular white matter. **(B)** All of the lesions show no enhancement on the CE T1WI. **(C–E)** Two of the lesions (arrows) in the posterior periventricular white matter are hypointense on the DWI, hyperintense on the ADC map, and hypointense on the eADC map, indicating chronic lesions with increased water diffusion due to severe tissue destruction.

Ten patients (10/22 = 45.5%) showed simultaneously asymptomatic enhancing and nonenhancing lesions on CE T1WI while 12 patients did not (mean enhancing lesions: 2.3 per person). All of the 51 enhancing lesions were hyperintense on DWI (type I lesion). Of the 51 enhancing lesions, one was hypointense on the ADC map and hyperintense on the eADC map (type IA lesion; Figure 1), while the other 50 lesions were isointense to hyperintense on the ADC map and isointense to hypointense on the eADC map (type IB lesion; Figure 2). Among the 333 nonenhancing lesions, 107 were hyperintense on DWI, iso- or hyperintense on the ADC map, and iso- or hypointense on the eADC map (type IIIB lesion; Figure 3), and 226 were nonhyperintense on DWI, iso- or hyperintense on the ADC map, and iso or hypointense on the eADC map (type IVB lesion; Figure 4). There were no type II, IIIA and IVA lesions. The results are summarized in Table 2. Hyperintensity on DWI was significantly linked to contrast enhancement on CE T1WI (*P* <0.001). The sensitivity, specificity, PPV, NPV, and accuracy for DWI to predict the enhancement of the demyelinating lesions on CE T1WI were 100%, 67.9%, 32.3%, 100%, and 72.1%, respectively.

**Table 2:** The numbers of 384 demyelinating lesions in each lesion type


**Lesion type**	IA	IB	IIA	IIB	IIIA	IIIB	IVA	IVB
**Lesion numbers**	1	50	0	0	0	107	0	226

Simultaneously asymptomatic enhancing and nonenhancing lesions (i.e. DIT) were present in 10 patients (45.5%) on CE T1WI while simultaneously hyperintense and nonhyperintense lesions were present in 12 patients (54.4%) on DWI (Table 3). In addition, the distribution of the demyelinating lesions in the aforementioned patients also met the MRI DIS criteria in 9 patients (40.9%) using CE T1WI and in 11 patients (50.0%) using DWI, respectively. Retrospectively, MS could be early diagnosed at CIS stage according to the 2010 Revisions to the McDonald Criteria in 40.9% patients using CE T1WI (and in 50.0% patients if DWI was used).

**Table 3:** Patients fulfilling MRI DIT criteria

**Used MRI sequence**	**Patient numbers**
CE T1WI	10 (45.5%)
DWI	12 (54.4%)

DIT, dissemination in time; CE T1WI, contrast-enhanced T1-weighted imaging; DWI, diffusion-weighted imaging.

## Discussion

MS is a chronic demyelinating disease of the CNS that usually has a relapsing-remitting course, and the heterogeneity of disease activity may be reflected in the clinical course, the immunopathological pattern as well as imaging appearance. Actually different stages of the disease process may be superimposed. The currently used McDonald Criteria require demonstration of dissemination of CNS demyelinating lesions in space and time, and the DIT criterion of the 2010 version can be fulfilled by a single MRI showing the simultaneous presence of asymptomatic gadolinium-enhancing and nonenhancing lesions in a patient at CIS stage. CE T1WI can distinguish active from inactive lesions in the brain since enhancement (corresponding to areas of active inflammation) occurs as a result of increased BBB permeability. One of the questions in the 2010 version of MRI DIT criterion is that the sensitivity is relatively low due to fewer asymptomatic enhancing brain lesions on a single baseline MRI. A Spanish cohort study revealed a 52.63% sensitivity [7] and our present study revealed that 45.5% patients fulfilled this criterion on a single baseline MRI. A more sensitive MR sequence to demonstrate DIT may be needed for possible early diagnosis of MS.

The contrast provided by DWI (unlike CE T1WI) depends on the molecular motion of water. Our study shows that enhancing lesions all have abnormal hyperintensity on DWI (100% sensitivity and 100% NPV) and type II lesion (enhancing on CE T1WI and nonhyperintense on DWI) is not found. However, many false positive type III lesions on DWI were not enhanced on CE T1WI. There are two possible reasons. First, the hyperintensity due to alterations of water diffusion in the lesions is more sensitive and lasts longer (may persist several months) than lesion enhancement due to transient BBB disruption (which usually lasts 4–6 weeks) [8-10]. Secondly, the high signal intensity of some lesions on DWI may be attributed to the “T2 shine-through” effect. The signal intensity on DWI is influenced by water diffusivity and the intrinsic T2 properties of the tissue being examined. The increased water content in demyelinating lesions may cause prolongation of T2 relaxation time and high signal on T2WI and thus, hyperintensity on DWI [9]. To remove the T2 shine-through effect, echo-planar spin echo T2WI (*b* = 0 sec/mm^2^) can be used to obtain an eADC map from DWI images. Our study shows that the T2 shine-through effect mainly exists in type IB and type IIIB lesions with hyperintensity on the DWI and hypointensity on the eADC map.

There were three imaging patterns of demyelinating lesions in our study. Type I lesions showed enhancement on CE T1WI that suggests active perivascular inflammation and BBB damage. Most active lesions have elevated diffusion resulting from disruption of myelin and increased extracellular space (vasogenic) edema (type IB) [11-13]. In rare instances, active lesions may reveal restricted diffusion with cytotoxic edema mimicking the radiological features of acute stroke (reduced ADC; type IA). It is likely to be an early stage of inflammation. The possible mechanisms of reduced ADC include infiltration of inflammatory cells (mainly T-lymphocytes and macrophages/microglial cells) and associated macromolecules as well as cytotoxic cell swelling, leading to reduced extracellular space [8-11,13-17]. The reduced ADC signal may take one to two weeks to revert to normal or increased signal [10,16,17]. CE T1WI may help to differentiate this early stage of demyelination from acute ischemic infarction because active demyelinating lesions usually enhance while acute ischemic infarcts do not [17]. In a recent study of DWI in 42 cases of atypical indiopathic inflammatory demyelinating lesions, restricted diffusion with low ADC values were seen in the outer enhancing part of the ring-like lesions, along the concentric rings of Balo-like lesions, and at the enhancing periphery of the infiltrative-type lesions, which corresponds to the areas of active inflammatory and demyelinating activities [18]. The lack of enhancement on CE T1WI in type IIIB lesions is likely due to recovery of the BBB. However, the persistent hyperintensity on DWI may suggest residual extracellular edema with increased diffusion, prolongation of T2 relaxation time, and the T2 shine-through effect. Type IVB lesions show elevated diffusion and no enhancement on CE T1WI that may be due to axonal loss and gliosis with widening of the extracellular space. They may even form “T1 black holes” on MRI, representing irreversible tissue destruction [10-12,14,19,20].

The limitations of this study are as follows: (1) The spatial resolution and signal-to-noise ratio of DWI were relatively suboptimal compared to SE/FSE imaging, so we had to exclude demyelinating lesions smaller than 3 mm in size. (2) Spinal cord lesions were not included in the study because DWI was not routinely used to evaluate the spinal cord in our study population. The magnetic field inhomogeneity around the spine, the small cross-sectional size of the spinal cord, and the increased motion in that area due to breathing, swallowing, and cerebrospinal fluid pulsation also limited its use. (3) The histopathology of different imaging types of demyelinating lesions was derived from previous studies (brain biopsy and autopsy evidence), and direct tissue proof was not obtained in our study. (4) The study was a retrospective one, and the interval between symptom onset and MRI studies varied (range: 1–44 days) and so may have influenced the results. A previous study showed that a better diagnostic performance for demonstration of simultaneous presence of gadolinium-enhancing and nonenhancing lesions was achieved when MRI was obtained within the first 30 days after symptom onset [4]. (5) The case number is relatively small.

## Conclusion

Our study have shown that lesion hyperintensity on DWI is significantly related to lesion enhancement on CE T1WI. We also delineated four types of demyelinating lesions (type IA, IB, IIIB and IVB) according to their status of enhancement on CE T1WI and signal intensity on DWI. Although DWI may not replace CE T1WI (the current gold standard) to demonstrate DIT because of the many false positive lesions, it may serve as a screening MRI sequence in cases where the use of GBCAs is a concern for its high sensitivity (100%). Future studies with tissue proof will be needed to prove if hyperintense and nonhyperintense demyelinating lesions on DWI could represent lesions in different stages of evolution (i.e. DIT).

## Abbreviations

MS: Multiple sclerosis; CNS: Central nervous system; DIS: Dissemination in space; DIT: Dissemination in time; MRI: Magnetic resonance imaging; CIS: Clinically isolated syndrome; BBB: Blood–brain barrier; GBCAs: Gadolinum-based contrast agents; DWI: Diffusion-weighted imaging; CE T1WI: Contrast-enhanced T1-weighted imaging; CDMS: Clinically definite multiple sclerosis; SE: Spin echo; FSE: Fast spin echo; T2WI: T2-weighted imaging; T2-FLAIR: T2-fluid-attenuated inversion recovery; ADC: Apparent diffusion coefficient; eADC: Exponential apparent diffusion coefficient; PPV: Positive predictive value; NPV: Negative predictive value.

## Competing interests

The authors have no competing interests to declare.

## Authors’ contributions

CPL was responsible for the design of the study and drafting the manuscript. All authors were responsible for data extraction, and CMC performed the statistical analysis. All authors have critically reviewed and approved the final version of the manuscript.

## Pre-publication history

The pre-publication history for this paper can be accessed here:

http://www.biomedcentral.com/1471-2377/14/100/prepub
